# Rickets, Vitamin D Deficiency and Tuberculosis in Children of St. Petersburg and its Region

**DOI:** 10.2174/0118743064436599260130204102

**Published:** 2026-02-04

**Authors:** Yulia Yarovaya, Marina Lozovskaya, Elenа Vasil'eva, Lyudmila Klochkova, Gennadij Stepanov, Ruslan Sagatelyan

**Affiliations:** 1 Department of Phthisiology, Saint Petersburg State Pediatric Medical University, Saint Petersburg, Russia

**Keywords:** Children, Vitamin D, Rickets, Tuberculosis infection, Tuberculosis, Nutrition

## Abstract

**Introduction:**

Given the widespread prevalence of vitamin D insufficiency and its clinical manifestations (such as rickets and compromised immune status) in children, this study aimed to assess the likelihood of tuberculosis (TB) diagnosis in children with TB infection and positive ESAT-6/CFP-10 test results, alongside signs of vitamin D deficiency (clinical rickets and/or low serum vitamin D levels).

**Method:**

A total of 98 children aged 1–14 years were examined at the TB department of St. Petersburg Children’s Infectious Diseases Hospital No. 3 (2022–2024). Inclusion criteria were a positive ATP test and/or IGRA result. Exclusion criteria included immunodeficiency disorders and hereditary diseases. Diagnostic evaluation for TB and vitamin D status (*via* serum calcidiol (25(OH)D) measurement was performed.

**Result:**

Rickets-related changes were observed in 70.4±4.6% of cases. Vitamin D insufficiency was detected in 21.4±4.4%, moderate deficiency in 36.7±5.1%, and severe deficiency in 33.7±5.0%. A significantly higher probability of active TB diagnosis was found in children with: • Rickets signs: OR=4.009 (95% CI 1.609–9.987), RR=1.872 (1.148–3.054), φ=0.310. • Vitamin D deficiency: OR=10.411 (3.762–28.809), RR=3.182 (1.648–6.145), φ=0.493. • Either factor: OR=41.167 (10.699–158.404), RR=27.986 (2.720–23.443), φ=0.677 (strong association).

**Discussion:**

The low vitamin D levels in children require not only special attention from TB specialists to patients with tuberculosis infection who test positive in immunological tests for ESAT-6 and CFP-10 antigens and show signs of rickets and/or vitamin D deficiency, but also increased vigilance from pediatricians in the timely diagnosis and treatment of rickets. Rickets often manifests early, has a prolonged course, and can lead to impaired immune status, which is significant for the progression from latent tuberculosis infection to active disease.

**Conclusion:**

Vitamin D deficiency and rickets should prompt heightened TB monitoring in high-risk children, emphasizing early pediatric intervention to prevent immunodeficiency and TB progression.

## 1 INTRODUCTION

Tuberculosis infection in children remains a relevant global public health issue to this day [1]. The incidence in the pediatric population is a prognostic indicator of tuberculosis burden in adulthood. Moreover, the proportion of undiagnosed tuberculosis cases globally for children aged 0 to 4 years is 58%, for ages 5 to 14 years, 45%, while for individuals aged 15 and older, it is 30% [2]. In the Russian Federation, the tuberculosis situation is improving, with a decline in key epidemic indicators [3]. To achieve the goals of further reducing the disease prevalence, optimizing the identification of tuberculosis risk groups is of great importance.

The activity of *M. tuberculosis* (Mycobacterium tuberculosis) can be determined by assessing the results of tests with ESAT-6 and CFP-10 antigens: the Tuberculin Allergen Recombinant (RTA) test and IGRA tests (QuantiFERON test or T-SPOT test), the positive results of which reveal the body's immune response to active *M. tuberculosis* [4, 5]. Children with immunodeficiency conditions constitute a risk group for tuberculosis [4]. Currently, a decrease in the number of children with combined tuberculosis and HIV infection is observed, while statistical monitoring for patients with other immunodeficiency conditions is lacking [3]. Children with immunodeficiency conditions, in case of infection with *M. tuberculosis*, showing signs of its activity as determined by positive results of immunological RTA or IGRA tests, are particularly susceptible to the risk of developing tuberculosis.

Vitamin D is a unique vitamin and steroid hormone [6-9]. Its first known function is participation in the formation and maintenance of the bone structure by regulating mineral metabolism (primarily calcium, phosphorus, and magnesium). Disruption of this metabolism leads to the development of rickets, which manifests already in early childhood [10-12]. The causal factors leading to vitamin D deficiency are diverse: insufficient dietary intake of this vitamin and minerals, as well as disorders in their absorption and/or excretion, hereditary and hormonal diseases [11, 12]. The clinical picture of rickets has common characteristic clinical features, and the duration of active disease spans from infancy to 3 or more years of age. After recovery, residual manifestations in the form of characteristic bone deformities often persist in preschool and early school-aged children, although laboratory deviations in mineral metabolism indicators from the norm are no longer observed, and bone tissue remineralization occurs relatively slowly [11, 12].

Currently, a broad spectrum of non-calcemic functions inherent to vitamin D has been proven [7, 8, 13-15]. One of its most significant roles in the body is participation in the induction and regulation of innate immunity [14, 16-18]. The active form of vitamin D – calcitriol (1,25-dihydroxyvitamin D) – influences virtually all cells in the body, including immune system cells, by acting on its specific VDR (vitamin D receptor) [7, 12, 19]. Calcitriol (1,25(OH)_2_D_3_) regulates the activity of all components of the delayed-type hypersensitivity reaction, which is the leading protective mechanism of the body against *M. tuberculosis*, and exerts immunomodulatory effects on cytokine and interferon secretion [19-23]. Importantly, calcitriol possesses an antimicrobial effect by inducing the production of biologically active peptides – cathelicidin and β-defensins – in cells with phagocytic activity (monocytes, macrophages, and neutrophils), which have a bactericidal effect on *M. tuberculosis* [24-26].

Currently, the dose-dependent effect of vitamin D on the immune system has been revealed, as well as the influence of calcidiol concentration in the body on the degree of VDR receptor expression, cytokine and interferon secretion [20, 22, 23], monocyte and macrophage activity, and local production of antimicrobial peptides (cathelicidin and β-defensins) by immune cells [25, 26]. The inactive form of vitamin D – calcidiol (25-hydroxyvitamin D) – thanks to its long half-life (about 2-3 weeks), is used to determine the body's vitamin D status by measuring its concentration in blood serum [6, 13].

A sufficient number of studies have been conducted, revealing the association between tuberculosis in adults and vitamin D levels [27-32]. There is a small number of literature sources dedicated to characterizing vitamin D concentration in children with different courses of tuberculosis infection. A study conducted in children with various courses of tuberculosis infection showed that in cases of Latent Tuberculosis Infection (LTBI) diagnosis, hypovitaminosis was noted in 58% of cases (level of 25-hydroxyvitamin D less than 50 nmol/l was considered insufficient), in children with active tuberculosis – in 75% of cases, and in patients of the control group (without tuberculosis infection) – in 43.3% of cases [33]. An analysis of vitamin D levels in children aged 2-17 years with various forms of tuberculosis revealed insufficiency in 13.3%, moderate deficiency in 34.7%, and severe deficiency in 49.3% of patients. In children with Latent Tuberculosis Infection (LTBI), the authors noted low vitamin D levels: moderate deficiency in 53% of cases, and insufficiency in the remaining children [34]. In children with LTBI aged 3-6 years, researchers found low concentrations of 25-hydroxyvitamin D in 87.5% of cases; in ages 7-17 years, in 96% of cases, including deficiency identified in 37.5% and 71% of children in these age groups, respectively [35]. In school-aged children receiving vitamin D, conversion of the tuberculin test to positive was observed in 11% of cases, which was less frequent compared to children receiving a placebo (27% of cases) [36].

Insufficient vitamin D status is widespread globally, including in the Russian Federation [37-39]. In a survey of residents of St. Petersburg, normal serum 25-hydroxyvitamin D were found in 17.9% of residents, its insufficiency was observed in 34.2%, and deficiency – in 47.9%; among the examined children and adolescents, only 6.7% had normal calcidiol levels, while 93.3% had serum 25-hydroxyvitamin D concentration corresponding to vitamin D deficiency and insufficiency [40]. In 2024, among residents of St. Petersburg of different genders and ages, vitamin D deficiency was identified in 48% of cases [41].

Given the high likelihood of insufficient vitamin D status in children, the broad spectrum of vitamin D's actions in the body, including its influence on mineral metabolism and the immune system, an analysis of the course of tuberculosis infection in children with clinical signs of active or past rickets and/or vitamin D deficiency is relevant.

The aim of the study was to determine the prognosis for diagnosing active forms of tuberculosis in children with tuberculosis infection and clinical signs of rickets and/or vitamin D deficiency.

## 2 METHODS

A prospective study of 98 children aged 1 to 14 years was conducted at the Tuberculosis Department of St. Petersburg Children's Infectious Diseases Hospital No. 3 during the period 2022–2024. The mean age of the children was 8.8±0.3 years, median – 9.0 years. Among the examined patients, 45.9% were boys, 54.1% were girls.

The inclusion criterion was a positive reaction of the RTA test and/or an IGRA test (TB-Feron test or QuantiFERON-TB test). Exclusion criteria were immuno-deficiency diseases and conditions, including HIV infection, immunosuppressive therapy, hereditary diseases, and, in some cases, early discharge of children at the parents' request before the diagnosis of tuberculosis and/or immunodeficiency disease was established. The examined patients had no chronic diseases or conditions affecting the intake, metabolism, or excretion of vitamin D.

The study design included two directions of diagnostic examination. The first direction was an in-depth examination for tuberculosis infection, which included: analysis of epidemiological history data, results of clinical examination, specific immunological diagnostics, spiral computed tomography (SCT) of the chest, bronchoscopic examination (when indicated), and bacteriological and molecular-genetic methods for detecting *M. tuberculosis* and its DNA.

During clinical examination, attention was paid to the possible presence of signs of active or past rickets, which are a consequence of impaired bone tissue mineralization. Criteria for residual rickets were characteristic bone deformities: flared lower chest aperture with protruding lower ribs, sternal depression (funnel chest), enlarged frontal skull bosses, in the absence of increased night sweating and other symptoms of the initial period of rickets (increased nervous excitability, hyperesthesia, increased vasomotor excitability). We observed these criteria in children aged 2 years and older. Criteria for active rickets were increased sweating in the occipital area during sleep, combined with soft bony edges of the fontanelle, or with enlarged frontal bosses, or with thickening of ribs at the costochondral junctions (“rachitic rosary”), which was observed in children in their first year of life.

Specific immunodiagnostics included performing and evaluating the RTA test, the Mantoux test, and, when indicated, the TB-Feron test or QuantiFERON-TB test. All children underwent MSCT of the chest.

The second direction of diagnosis was determining vitamin D status, which was carried out by measuring the concentration of 25-hydroxyvitamin D in blood serum using a solid-phase enzyme-linked immunosorbent assay (ELISA). For this purpose, blood was drawn from children in the morning on an empty stomach. Assessment of vitamin D content in the body was conducted depending on the detected concentration of 25-hydroxyvitamin D in serum in accordance with the recommendations of the National Program “Vitamin D Deficiency in Children” [8]: insufficiency was assessed at a concentration of 20 to 30 ng/ml (50 to 75 nmol/l); moderate deficiency – concentration less than 20 ng/ml down to 10 ng/ml (or less than 50 nmol/l down to 25 nmol/l); severe (pronounced, avitaminosis) vitamin D deficiency – less than 10 ng/ml (or less than 25 nmol/l).

To characterize mineral metabolism, disturbances of which are observed in active rickets, the content of calcium, phosphorus, and magnesium in patient serum was determined using a colorimetric method. The reference intervals for normal concentration values of these mineral elements were: for calcium – 2.2-2.65 mmol/l, phosphorus – 0.81-1.45 mmol/l, magnesium – 0.77-1.03 mmol/l.

The parents or guardians of these patients signed a voluntary Informed Consent form for participation in the study upon admission to the department (facilitated by the Legal Department of the St. Petersburg State Pediatric Medical University). The study was approved by the decision of the Local Ethics Committee at the St. Petersburg State Pediatric Medical University (Protocol No. 17/03 dated 15.12.2023), and complies with international ethical standards set forth in the World Medical Association's Declaration of Helsinki “Ethical Principles for Medical Research Involving Human Subjects”.

Based on the results of phthisiatric diagnostics, two patient groups were identified: Group 1 – 60 children with active tuberculosis (61.2% of cases), Group 2 – 38 children with latent tuberculosis infection (LTBI, 38.8% of cases).

**Diagnostic criteria for active tuberculosis were:** positive results of the RTA test and/or IGRA tests; detection on chest SCT of chest organ changes characteristic of tuberculosis; positive results of micro-biological, bacteriological, and PCR diagnostics of patient samples reliably confirmed tuberculosis, although negative results of these tests did not rule it out.

**Diagnostic criteria for latent tuberculosis infection (LTBI) were:** positive results of the RTA test and/or IGRA tests; absence of chest organ changes or non-specific changes on chest SCT that resolved after non-specific antibacterial therapy; negative results of microbiological, bacteriological, and PCR diagnostics of patient samples.

Statistical analysis was performed using specialized StatSoft STATISTICA software and included the assessment of laboratory parameters using descriptive statistics methods. Results are presented as arithmetic means with standard error of the mean (M±m). Quantitative indicators were assessed for normality of distribution using the Kolmogorov-Smirnov test (for n≥50) and the Shapiro-Wilk test (for n<50). For the assessment of categorical variables, analysis of four-field contingency tables was performed with calculation of odds ratio (OR) and risk ratio (RR) indicators with a 95% confidence interval, the two-sided Fisher's exact test, Pearson's χ2 test, or for n<10, χ2 with Yates' correction (at a significance level of α=0.05). The strength of association between categorical variables was assessed using Cramér's V (φc) coefficient. To determine the significance of differences between the compared mean values of the analyzed groups, Student's t-test was used (differences were considered statistically significant at *p*<0.05).

## 3 RESULTS

The vast majority of patients were vaccinated against tuberculosis - in 94.9% of cases; in Group 1 – 93.3%, in Group 2 – 97.4%. Tuberculosis contact was established in 39.8% of patients; in Group 1 – 50.0%, in Group 2 – 23.7%. Sputum positivity (bacteriologically confirmed source) was identified in 82.1% of the infection sources for the children (n = 39), among which drug resistance of *M. tuberculosis* was determined in 43.8% of cases (n = 32).

The examined children were, in most cases, natives of St. Petersburg and its (Leningrad) region – 69.4% of cases (in Group 1, 68.3% of cases; in Group 2, 71.1% of cases); the remaining patients were from other regions who had been residing in St. Petersburg for 2 years or more.

At the time of examination, the RTA test was positive in 94.9% of patients; 5 patients with a negative result (5.1% of cases) had a positive TB-Feron test (one patient in Group 1 and four children with LTBI), reflecting infection with signs of *M. tuberculosis* activity.

Analysis of the dynamics of immunological tests (Mantoux test and RTA test) prior to hospitalization showed that the duration of *M. tuberculosis* infection of up to 1 year was observed in 66.3% of the examined children (in Group 1 – 66.7%, in Group 2 – 65.8%), including infection duration of up to 6 months in 59.2% of patients (in Group 1 – 60.0%, in Group 2 – 57.9%). An infection duration of more than one year was noted in 20.4% of children (in Group 1 – 23.3%, in Group 2 – 15.8% of patients). In a number of cases (13.2%), determining the time of infection was difficult due to young age or incomplete dynamics of immunological tests (in Group 1 – 6.1%, in Group 2 – 18.4% of children).

Children with active tuberculosis predominantly presented with clinical forms of the primary period; the structure of clinical forms is presented in Table **1**.

The most frequent complications were foci of dissemination (seeding) into the lung tissue, observed in 28.3% of children, including in combination with bronchopulmonary lesions, exudative pleurisy, and atelectasis (one case each). Less frequently diagnosed were: bronchial tuberculosis – in 3.3% of cases, bronchopulmonary lesions and encapsulated pleurisy – in 1.7% of cases each.

Diagnosis of the specific process occurred in the phase of incomplete calcification in 55.0% of patients, less frequently in other phases: infiltration – in 30.0%, infiltration and beginning calcification – in 8.3%, infiltration and disintegration (cavitation) – in 5.0%, and exudation – in 1.7% of children. Sputum positivity (bacteriological confirmation) was detected in 6.7% of patients, among whom drug resistance of *M. tuberculosis* was determined in 5.0% of cases (n=60).

Results of the clinical examination upon admission revealed residual changes after past rickets in the form of bone deformities in the majority of children (68.4±4.7% of cases); in two young children, manifestations of the peak period were observed. The data are presented in Table **2**.

Clinical signs of rickets were observed significantly more often in patients with active forms of tuberculosis (81.7±4.5% of cases) than in children with LTBI – 52.6±8.1% of cases. Some children exhibited combinations of signs of past rickets: in Group 1 children, a combination of flared chest aperture and sternal deformity in 28.3±5.8%, leg bowing in 6.7±3.2%, and large frontal bosses in 1.7±1.7% of cases; in Group 2 children – a combination of flared aperture with sternal deformity in 10.5±4.9%, and with leg bowing in 2.6±2.6% of cases.

Considering that the development of rickets begins in early childhood and continues for several years, and the duration of infection in the majority of children was up to one year (66.3% of patients), the examined patients were characterized by a more prolonged state of low vitamin D status compared to the duration of exposure to actively metabolizing *M. tuberculosis*.

The results of the examination of serum 25-hydroxyvitamin D concentration indicated low vitamin D status in 91.8±2.9% of all examined children. Vitamin D insufficiency was present in 21.4±4.4% of patients, and deficiency in 70.4±4.9% of children, including moderate deficiency in 36.7±5.1% and severe deficiency in 33.7±5.0% of those examined.

Comparison of vitamin D status between patients with active tuberculosis and children with LTBI showed that in children with LTBI, normal vitamin D levels (10.0±3.9% of cases) and insufficiency (10.0±3.9% of cases) were observed less frequently than in patients with active tuberculosis (18.4±6.3% of cases and 39.5±7.9% of cases, respectively). Conversely, vitamin D deficiency was more frequently diagnosed in patients with active tuberculosis – in 88.3±4.2% of cases compared to patients with LTBI (42.1±8.0% of cases, Table **3**).

The mean concentration of serum 25-hydroxyvitamin D of all children with tuberculosis infection was 15.28±0.87 ng/ml, corresponding to the grade of moderate deficiency.

Despite the low vitamin D status of the overwhelming majority of examined children, the mean content of calcium and magnesium was within normal limits (2.45±0.03 and 0.86±0.01 mmol/l, respectively), indicating in favor of the completion of the active phase of rickets by the time of examination. However, the phosphorus content was moderately elevated (1.67±0.02 mmol/l, Table **4**).

Serum phosphorus content was moderately elevated in the majority of patients (57.1±5.1% of cases); in Group 1 patients in 43.3±6.4% of cases, and less frequently in Group 2 in 78.0±6.7% of cases (*p*=0.0031). This can be explained both by an increased release of these mineral elements from tissues due to their necrobiotic damage during the tuberculous process and by ongoing processes of exacerbation and convalescence of rickets: bone resorption, which leads to increased serum levels of both calcium and phosphorus, and its subsequent reminerali-zation. Normal calcium (in 90.8±2.9% of cases) and magnesium (in 88.8±3.2% of cases) content predominated in the patients.

Analysis of the frequency of tuberculosis diagnosis in children infected with *M. tuberculosis* (positive results of the RTA tests and/or IGRA tests) and in whom the following signs of low vitamin D status were identified: signs of rickets, diagnosis of vitamin D deficiency at the time of examination (reduced concentration of 25-hydroxyvitamin D less than 20 ng/ml), as well as the identification of one and/or both of these signs, deter-mined a greater prognostic likelihood of active tuber-culosis in case of detecting any of the aforementioned signs (Table **5**).

Thus, the highest frequency of active tuberculosis diagnosis was in cases where children with tuberculosis infection (positive RTA/or IGRA tests) had any of the signs of low vitamin D status: clinical signs of rickets and/or deficiency of this vitamin (strong association strength - φ 0.677).

## 4 DISCUSSION

To date, there are sufficient studies revealing the mechanisms of induction and regulation of innate immunity carried out by vitamin D. Calcitriol affects all components of delayed-type hypersensitivity: it participates in the activation of macrophages and lymphocytes, regulates the synthesis of interferons and cytokines by immune cells, and, most importantly, initiates the synthesis of biologically active peptides cathelicidin and β-defensins by macrophage cells, which have a bactericidal effect on *M. tuberculosis*. On the other hand, the course of active tuberculosis can also influence the absorption and metabolism of vitamin D. However, considering that the examined children with tuberculosis were, in most cases, infected with *M. tuberculosis* within one year, the diagnosis was made promptly before the development of overt clinical symptoms, and children with LTBI had no clinical or radiological manifestations of the disease, we considered the influence of vitamin D on the course of tuberculosis infection to be predominant.

Categorization of vitamin D was based on the National Program “Vitamin D deficiency in children” (M.: Pediatrician; 2018), which does not account for the variability of laboratory indicators, seasonality, or the influence of the child's age, except for preterm newborns, who were absent in our study. This categorization is defined according to the recommendations of Holick *et al*. [13]. There are disagreements regarding the interpretation of threshold concentrations of 25(OH)D: a number of international bodies recommend maintaining levels above 20 ng/ml (50 nmol/l) [10, 13], while other experts recommend levels above 30 ng/ml (75 nmol/l) [40]. It is interesting to note that the Vitamin D for the Prevention of Disease: An Endocrine Society Clinical Practice Guideline suggests empiric vitamin D for those aged 1 to 18 years and adults over 75 years of age, those who are pregnant, and those with high-risk prediabetes. The panel suggests against routine 25(OH)D testing in the absence of established indications [41].

Residual signs of past rickets indicated a prolonged state of low vitamin D status over several years, and consequently, its long-term insufficient influence on the child's immune development, which is significant for immune protection against *M. tuberculosis*. Given that rickets is a prolonged disease lasting several years, it correspondingly exerts a long-term negative influence on the child's developing immunity. Therefore, we combined the signs of active and residual rickets, as both reflect insufficient functioning of the immune system.

Normal vitamin D levels were observed in 8.2% of all examined children, which is close to the value noted in a literature source indicating 6.7% of children and adolescents in St. Petersburg with normal vitamin D status [42]. However, the results of our study revealed a smaller number of patients with normal vitamin D content among children with active tuberculosis (1.7% of cases) compared to children with LTBI (18.4% of cases with normal vitamin D status). Furthermore, there was a difference in the number of patients with identified vitamin D deficiency: among residents of St. Petersburg, regardless of sex and age, deficiency was observed in 48% of cases [43], while according to our study results – in 70.4% of cases. Among the examined patients with active tuberculosis, children with vitamin D deficiency were more numerous (88.3% of cases) compared to children with LTBI (42.1% of cases).

Our study did not examine the influence of many concomitant factors (nutrition, socioeconomic status, sunlight exposure) because the examined children were not diagnosed with nutritional disorders or diseases affecting the intake, metabolism, or excretion of vitamin D, and sunlight exposure for all residents of St. Petersburg is comparable (all children lived in St. Petersburg either since birth or for a period of at least two years). The socioeconomic status of the patients could affect the quality of nutrition, which is significant both for the development of vitamin D deficiency and the risk of developing tuberculosis. Since a larger number of patients is required for multivariate analysis, we will account for these influences in further studies, which should examine a larger patient cohort necessary for statistical processing in multivariate analysis.

The results of our study confirm the immunological role of vitamin D in reducing immunity and, consequently, in provoking the development of tuberculosis in children infected with *M. tuberculosis* with positive results of immunological tests for ESAT-6 and CFP-10 antigens and the presence of signs of rickets and/or diagnosis of vitamin D deficiency. We have determined a threshold level for the reduction of 25-Hydroxyvitamin D Concentration (less than 20 ng/ml) that has practical significance as a risk factor for tuberculosis development in these patients. Therefore, timely diagnosis of rickets, vitamin D deficiency, and their subsequent treatment in childhood can reduce the incidence of tuberculosis in children, and consequently, reduce the reservoir of tuberculosis in childhood and its reactivation in adulthood.

## CONCLUSION

1. Clinical signs of rickets in the form of bone deformities were more frequently identified in children with active tuberculosis (81.7±4.5% of cases) than in children with LTBI (52.6±8.1% of cases, *p*=0.0029).
2. Normal vitamin D status (1.7±1.7% of cases) and vitamin D insufficiency (10.0±3.9% of cases) were observed more often in children with LTBI than in patients with active tuberculosis (18.4±6.3% of cases, *p*=0.012, F=0.0041, χ2 _Yates_=6.620) and (39.5±7.9% of cases, *p*=0.0012, F=0.00085, χ2 _Yates_=10.317), respectively. Conversely, vitamin D deficiency predominated in patients with active tuberculosis (88.3±4.2% of cases) compared to children with LTBI (42.1±8.0% of cases, *p*<0.0001, F=0.000, χ2 _Yates_=21.696).
3. A higher likelihood of tuberculosis diagnosis was established in children infected with *M. tuberculosis* with positive ESAT-6 and CFP-10 antigen tests when the following were identified: signs of rickets (OR=4.009 CI [1.609-9.987] SE=0.466 RR=1.872 CI [1.148-3.054] SE=0.250 φ=0.310), vitamin D deficiency at the time of examination (OR=10.411 CI [3.762-28.809] SE=0.519 RR=3.182 CI [1.648-6.145] SE=0.336, φ=0.493), or the presence of one or both of these signs (OR=41.167 CI [10.699-158.404] SE=0.688 RR=27.986 CI [2.720-23.443] SE=0.549 φ=0.677).

## Figures and Tables

**Table 1:** Structure of clinical forms of active tuberculosis in the examined children.

**Clinical Form of Tuberculosis**	**Number of Patients**	**% of Cases**
Uncomplicated forms of intrathoracic localization, including	35	58,3
• Tuberculosis of intrathoracic lymph nodes	13	21,7
• Primary tuberculous complex	15	25,0
• Tuberculous pleurisy	1	1,7
• Focal tuberculosis	4	6,7
• Infiltrative tuberculosis	2	3,3
Generalized tuberculosis	4	6,7
Complicated forms of intrathoracic localization, including	21	35,0
• Tuberculosis of intrathoracic lymph nodes	18	30,0
• Primary tuberculous complex	2	3,3
• Focal tuberculosis	1	1,7
Total	60	100

**Table 2:** Clinical signs of rickets in the examined children.

**Signs of Rickets in Children**	**Children Examined with Tuberculosis Infection**					
	**Group 1: Children with Active Tuberculosis,** **n = 60**		**Group 2: Children with LTBI,** **n = 38**		**All Examined Patients,** **n = 98**	
	**Abs.**	**%**	**Abs.**	**%**	**Abs.**	**%**
Residual changes after rickets, including	47	78,3±5,3 *p*= 0,0094*	20	52,6±8,1 *p* =0,0094*	67	68,4±4,7
▪ Harrison's groove	45	75,0±5,6 *p*= 0,00041*	15	39,5±7,9 *p*= 0,00041*	60	61,2±4,9
▪ Deformation of the sternum	19	31,7±6,0 р= 0,134	7	18,4±6,3 *p*= 0,134	26	36,5±4,8
▪ Curvature of the lower extremities	4	6,7±3,2 *p*= 0,325	1	2,6±2,6 *p*= 0,325	5	5,1±2.2
Active changes: enlarged frontal tubercles combined with increased sweating	2	3,3±2.3	-	-	2	2,0±1,4
Total	49	81,7±4,5 *p*= 0,0029*	20	52,6±8,1 *p*= 0,0029*	69	70,4±4,6

Note: *statistical significance of differences in indicators between groups 1 and 2 at *p*<0.05.

**Table 3:** Vitamin D status in children with tuberculosis infection.

**Vitamin D Status in accordance with the 25-hydroxyvitamin D Concentration**	**Children Examined with Tuberculosis Infection**					
	**Group 1: Children with Active Tuberculosis,** **n = 60**		**Group 2: Children with LTBI,** **n = 38**		**All Examined Patients,** **n = 98**	
	**Abs.**	**%**	**Abs.**	**%**	**Abs.**	**%**
Normal vitamin D status - 30 ng/ml and above	1 F=0,0051* χ^2^_Yates_ =6,620*	1,7±1,7 *p*=0,012*	7 F=0,0041* χ^2^_Yates_ =6,620*	18,4±6,3 *p* =0,012*	8	8,2±2,9
Insufficiency - less than 30 to 20 ng/ml	6 F=0,00085* χ^2^_Yates_ =10,317*	10,0±3,9 *p* =0,0012*	15 F=0,00085* χ^2^_Yates_ =10,317*	39,5±7,9 *p*=0,0012*	21	21,4±4,4
Deficiency - less than 20 ng/ml, including:	53 F=0,000* χ^2^_Yates_ =21,696*	88,3±4,2 *p* < 0,0001*	16 F=0,000* χ^2^_Yates_ =21,696*	42,1±8,0 *p* < 0,0001*	69	70,4±4,9
Moderate - less than 20 ng/ml to 10 ng/ml	27 F=0,052 χ^2^_Yates_ =3,678	45,0±6,4 *p* = 0,026*	9 F=0,052 χ^2^_Yates_ =3,678	23,7±6,9 *p* =0,026*	36	36,7±5,1
Severe - less than 10 ng/ml	26 F=0,015* χ^2^_Yates_ =5,398	43,3±6,4 *p* =0,0065*	7 F=0,015* χ^2^_Yates_ =5,398	18,4±6,3 *p* =0,0065*	33	33,7±5,0
Total	60	100	38	100	88	100

Note: *statistical significance of differences in indicators between groups 1 and 2 at *p*<0.05.

**Table 4:** Indicators of мineral elements content in the examined children.

**Мineral Elements**	**Mean Content of Serum Mineral Elements, mmol/L, M±m**			**Reference Intervals,** **ng/ml**
	**1 Group, n=60,** **M ± m, ng/ml**	**2 Group, n=38** **M ± m, ng/ml**	**All Patients,** **n=98, M ± m, ng/ml**	
Calcium	2,39±0,05	2,51±0,05	2,45±0,03	2,2-2,65
Magnesium	0,85±0,01	0,87±0,01	0,86±0,01	0,77-1,03
Phosphorus	1,65±0,03	1,69±0,03	1,67±0,02	0,81-1,45

**Table 5:** Course of tuberculosis infection in children with positive reactions to ESAT-6 and CFP-10 antigens in the presence of signs of insufficient vitamin D status: Manifestations of rickets, reduced concentration of 25-hydroxyvitamin D less than 20 ng/ml (vitamin D deficiency), and identification of one or both of these signs.

**Course of Tuberculosis Infection/signs of Reduced Vitamin D Sufficiency**	**Active Tuberculosis Abs. (%)**	**LTBI Abs. (%)**	**F**	**χ^2^**
Presence of rachitic changes, n = 69	49 (71,0%)	20 (29,0%)	0,003*	χ^2^ =9,414*
Absence of rachitic changes, n = 29	11 (37,9%)	18 (62,1%)	0,003*	χ^2^ =9,414*
OR=4,009 CI [1,609*-* 9,987] SE = 0,466 RR = 1,872 CI [1,148- 3,054] SE = 0,250 φ 0,310				
Concentration of 25-Hydroxyvitamin D less than 20 ng/ml (vitamin D deficiency), n = 61	53 (86,9%)	16 (26,2%)	0,000*	χ^2^_yates_ =21, 696*
Concentration of 25-Hydroxyvitamin D 20 ng/ml and above (insufficiency and normal vitamin D status), n=27	7 (25,9%)	22 (81,5%)	0,000*	χ^2^_yates_=22, 696*
OR= 10,411 CI [3,762*- 2*8,809] SE= 0,519 RR=3,182 CI [1,648- 6,145] SE=0,336 φ 0,493				
Presence of signs of rickets and/or vitamin D deficiency, n=69	57 (82,6%)	12 (17,4%)	0,000*	χ^2^_yates_=41,922*
Absence of combined presence of these factors, n=29	3 (10,3%)	26 (89,7%)	0,000*	χ^2^_yates_=41,922*
OR= 41,167 CI [10,699*-*158,404] SE= 0,688 RR=27,986 CI [2,720-23,443] SE= 0,549 φ=0,677				

Note: *statistical significance of differences in indicators between groups at *p*<0.05.

## Data Availability

All data generated or analyzed during this study are included in this published article.

## LIST OF ABBREVIATIONS

VDR: Vitamin D receptor
LTBI: Latent Tuberculosis Infection
SCT: Spiral Computed Tomography
ELISA: Enzyme-Linked Immunosorbent Assay
OR: Odds Ratio
RR: Risk Ratio

## STANDARDS OF REPORTING

STROBE guidelines were followed.
